# Commentary on the T1D Exchange Quality Improvement Collaborative Learning Session November 2024 abstracts

**DOI:** 10.1111/1753-0407.70037

**Published:** 2024-12-25

**Authors:** Halis K. Akturk, Osagie Ebekozien, Holly Hardison, Nicole Rioles, Stephanie Crossen

**Affiliations:** ^1^ Barbara Davis Center for Diabetes University of Colorado Aurora California USA; ^2^ T1D Exchange Boston Massachusetts USA; ^3^ University of Mississippi School of Population Health Jackson Mississippi USA; ^4^ University of California, Davis Sacramento California USA

## Abstract

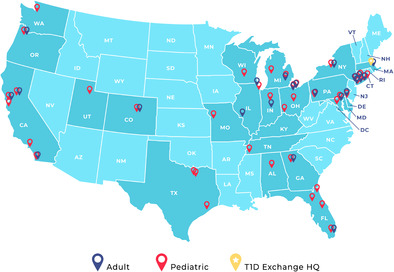

The T1D Exchange is a nonprofit organization dedicated to accelerating therapies and improving care for individuals with type 1 diabetes. Its Quality Improvement Collaborative (T1DX‐QI) comprises a network of 62 adult and pediatric diabetes centers (Figure 1), which collectively care for more than 250 000 people with diabetes (PWD). The T1DX‐QI harnesses real‐world data from its centers to evaluate and improve the quality of care delivered nationally to PWD, and its annual T1DX‐QI Learning Session highlights the depth and breadth of this work.

**FIGURE 1:**
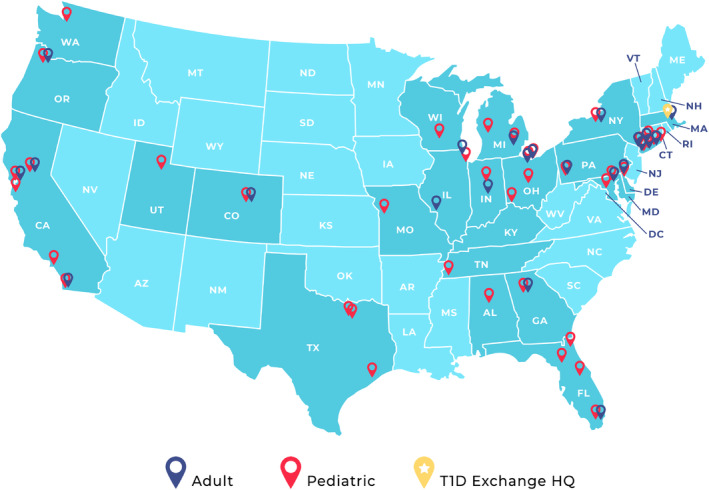
Map of centers in the T1DX‐QI.

The 2024 Learning Session covered many important and timely topics, including the uptake of diabetes technologies, programs to support pediatric‐to‐adult transition of care, novel educational tools and approaches, strategies to identify and address social drivers of health (SDOH), and optimizing access to and outcomes of care for PWD populations. The overall focus of the conference, in keeping with the mission of the T1DX‐QI, was on reducing health disparities and promoting equitable care.

Many abstracts detailed centers' efforts to improve screening for diabetes comorbidities, including hyperlipidemia (Sing et al.), microalbuminuria (Holly et al.), and retinopathy (Ilkowitz et al.). Centers also reported on their efforts to improve identification and management of comorbid mental health conditions. Zimmerman et al. implemented automated EMR eating disorder screening in a pediatric population with type 1 diabetes and identified that 17% of those screened were at risk for an eating disorder. Wolf et al. implemented ASQ suicide risk screening in routine pediatric diabetes care and found that suicide risk was higher in youth with type 2 diabetes compared to those with type 1 diabetes, and that the PHQ‐9 was less sensitive in identifying suicide risk in adolescents and young adults compared to the ASQ. Jimenez et al. improved depression screening rates among adolescents with type 1 diabetes from <30 to >70% by implementing an annual in‐person screening visit with supplemental staffing and workflows.

In the realm of diabetes education, abstracts reported the utility of standardized approaches both for inpatient nursing diabetes education (Gunckle et al.) and documenting insulin pump back‐up plans for patients (Jones et al.). Moore et al. surveyed providers caring for children with diabetes on ages at which patients should master certain diabetes topics and skills. Surveyed providers reported an unmet need for official guidelines on this topic as well as systems to evaluate and track achievement of these milestones by patients. In a similar vein, Schmitt et al. compared expectations of families and of diabetes educators regarding the readiness of children and adolescents to independently manage insulin injections, self‐monitoring of glucose levels, and carbohydrate counting. Surprisingly, 20% of surveyed families felt a 6‐year‐old could check blood glucose levels without supervision and 21% expected children younger than age 10 to count carbohydrates independently, highlighting the need to develop educational tools related to age‐appropriate diabetes care tasks.

The use of diabetes technology was the focus of numerous abstracts at this year's Learning Session. Multiple centers reported their QI efforts to improve early adoption of technology after T1D diagnosis through improvements in device education and prescribing practices (Milosavljevic et al.), improved support after prescription (Perkins et al.), or both (Leverenz et al., Vakharia et al., Garrity et al.). Several focused specifically on populations such as patients with HbA1c >8.5% (Medina et al); PWD with non‐English language preferences (Goldklang et al.), and many showed impressive results, increasing AID use in recently diagnosed patients from 1% to 30% (Vakharia et al.) or doubling insulin pump utilization among non‐English language preferring patients (Goldklang et al.).

Abstracts about interventions supporting PWD with elevated HbA1c levels—described the importance of an individualized, proactive, multidisciplinary and person‐centered approach (Ilkowitz et al., Kelly et al., Odugbesan et al). Muthuvel et al. employed a mobile care center to bring in‐person care closer to homes for youth with type 1 diabetes and SDOH needs; they noticed a dramatic HbA1c reduction from 13.5 to 6.5% over two visits after initiating an AID system. Many centers also reported on their efforts to improve support for transitions from pediatric to adult care, noting the utility of standardized elements such as a transition curriculum, a focused transition clinic, and a transition visit checklist (Rosenheck et al., Ross et al., Grundman et al., Lavik et al., Mojica et al.).

Several centers' abstracts focused on health services and healthcare costs. Waterman et al. characterized the impact of insulin costs on health among 146 pediatric PwT1D, among whom higher monthly insulin costs was associated with insulin rationing behavior and higher HbA1c. Harris et al. detailed the design and launch of the first pediatric specialty value‐based program for PwT1D, while Howell et al. developed a self‐service dashboard that integrates and transforms data from multiple sources, including EMR, to enable effective visualization and tracking of population changes over time. And two abstracts demonstrated the impact of QI efforts on non‐insulin medication use overall (Vakharia et al.) and GLP1 agonist use specifically (Huang et al.) among youth with type 2 diabetes.

The 2024 T1D Exchange Quality Improvement Collaborative Learning Session (Table S1) showcased the outstanding work being done by T1DX‐QI centers to define existing gaps in care and advance effective tools to address them. Moving forward, the Collaborative will continue to focus on QI innovations that improve access to quality diabetes care for underserved, minoritized and populations with chronically elevated HbA1c and promote health equity for all people living with diabetes.

## Supporting information


**Supplemental Table S1.** T1DX‐QI November Learning Session Planning Committee.

