# Supplementary material for: Commentary on the T1D Exchange Quality Improvement Collaborative Learning Session November 2024 abstracts
Source: J Diabetes. 2024 Dec 25;16(12):e70037. doi: 10.1111/1753-0407.70037 (PMC11669477; doi:10.1111/1753-0407.70037)
Supplement: Supplementary file 1 — Supplemental Table S1. T1DX‐QI November Learning Session Planning Committee. [file JDB-16-e70037-s001.docx]

Supplemental Table 1: T1DX-QI November Learning Session Planning Committee

| **Member Name** | **Member Role** | **Member Affiliation** |
| --- | --- | --- |
| *T1DX-QI National Committee Members* | | |
| Ashley Butler, MD | Health Equity Advancement Lab (HEAL) Committee Co-Chair | Baylor College of Medicine, Section of Pediatric Diabetes and Endocrinology, Houston, TX |
| Blake Adams, BSN, RN | QI Champion Committee Co-Chair | University of Tennessee Health Science Center, Le Bonheur Children's Hospital Memphis, TN |
| Carla Demeterco- Berggren, MD, PhD | Clinical Leadership Committee Co-Chair | Rady Children's Hospital, San Diego, CA |
| David Hansen, MD, MPH | Data Governance Committee Co-Chair | SUNY Upstate Medical University, Syracuse, NY |
| David Maahs, MD, PhD | International Collaboration Committee Chair | Stanford University School of Medicine, Stanford, CA |
| Emily Coppedge, NP | People with Diabetes (PWD Advisory Committee Co-Chair | Weill Cornell Medicine, New York, NY |
| Francesco Vendrame | Clinical Leadership Committee Co-Chair | University of Miami Miller School of Medicine, Miami FL |
| Georgia Davis, MD | Data Governance Committee Co-Chair | Emory University School of Medicine Division of Endocrinology, Atlanta, GA |
| Halis K. Akturk | Publication Committee Co-Chair | Barbara Davis Center for Diabetes, University of Colorado, Aurora, CA |
| Jeniece Ilkowitz, RN, MA, CDCES | People with Diabetes (PWD Advisory Committee Co-Chair | Pediatric Diabetes Center, NYU Langone Health, New York, NY, |
| Lydia Holly, BSN, RN | QI Champion Committee Co-Chair | Children's National Hospital, Washington, DC |
| Nirali Shah, MD | Data Science Committee Co-Chair | Icahn School of Medicine at Mount Sinai, New York, NY |
| Ryan McDonough, DO, FAAP | Data Science Committee Co-Chair | Children’s Mercy Hospital, Kansas City, MO |
| Selorm Dei-Tutu, MD, MPH | Health Equity Advancement Lab (HEAL) Committee Co-Chair | Baylor College of Medicine, Section of Pediatric Diabetes and Endocrinology, Houston, TX |
| Stephanie Crossen, MD, MPH | Publication Committee Co-Chair | University of California, Davis, Sacramento, CA |
| *T1DX-QI Coordination Center Members* | | |
| Anton Wirsch, MS | Lead, Data Management | T1D Exchange, Boston, MA |
| Dhruvi Vora, BS | Analyst, Real World Data | T1D Exchange, Boston, MA |
| Don Buckingham, Sr., MBOE, CPHQ | Senior QI Consultant | T1D Exchange, Boston, MA |
| Emma Ospelt, MPH | Senior Data Analyst, Real World Data | T1D Exchange, Boston, MA |
| Holly Hardison, BS | QI Analyst | T1D Exchange, Boston, MA |
| James Dawson, BS | Data Integration Analyst | T1D Exchange, Boston, MA |
| Nicole Rioles, MA | Senior Director of Clinical Partnerships | T1D Exchange, Boston, MA |
| Ori Odugbesan, MD, MPH | Associate Director of QI and Health Equity | T1D Exchange, Boston, MA |
| Osagie Ebekozien, MD, MPH | Chief Medical Officer | T1D Exchange, Boston, MA |
| Saketh Rompicherla, MS | Population Health Analyst | T1D Exchange, Boston, MA |
| Susan Thapa, PhD, MPH | Associate Director Real World Data | T1D Exchange, Boston, MA |
| Timothy Bol, BA | QI Analyst | T1D Exchange, Boston, MA |
| Trevon Wright, MHA | Manager, Quality Improvement and Special Projects | T1D Exchange, Boston, MA |
